# Supplementary material for: Empirical methods for controlling false positives and estimating confidence in ChIP-Seq peaks
Source: BMC Bioinformatics. 2008 Dec 5;9:523. doi: 10.1186/1471-2105-9-523 (PMC2628906; doi:10.1186/1471-2105-9-523)
Supplement: Additional File 1 — A variety of html documents from the USeq web site detailing the available applications, their best usage, output file type descriptions, command line menus, etc. [file 1471-2105-9-523-S1.zip › USeqUserGuides/JavaDoc.zip!/JavaDoc/util/bio/cluster/HierarchicalClustering.html]

HierarchicalClustering


|  |  |  |
| --- | --- | --- |
| **Overview** \| **Package** \| **Class** \| **Use** \| **Tree** \| **Deprecated** \| **Index** \| **Help** | |  |
| **PREV CLASS**   NEXT CLASS | **FRAMES**    **NO FRAMES**     **All Classes** |  |
| SUMMARY: NESTED \| FIELD \| CONSTR \| METHOD | DETAIL: FIELD \| CONSTR \| METHOD |  |


---


## util.bio.cluster Class HierarchicalClustering

```
java.lang.Object
  util.bio.cluster.HierarchicalClustering
```

---

``` public class HierarchicalClustering extends java.lang.Object ```

Calculates a Pearson correlation coefficient between all pairs of serialized float[]s, uses hierarchical clustering to
group like arrays. Displays on screen and saves as a png.

---

**Constructor Summary**

`HierarchicalClustering(double minRValue,
int maxSizeToFlagCluster,
java.lang.StringBuffer results,
java.io.File pngResultFile,
boolean displayClusterPlot)`
  
          For integration with CelFileQualityControl App.

`HierarchicalClustering(java.io.File[] floatArrayFiles)`
  
          For integration with Correlate App.

`HierarchicalClustering(java.io.File[] floatArrayFiles,
java.io.File pngResultFile)`
  
          For integration with CelProcessor.

`HierarchicalClustering(java.lang.String[] args)`
  
          For stand alone use.


| **Method Summary** | |
| --- | --- |
| `void` | `breakOutClusters()` |
| `void` | `cluster()` |
| `java.io.File[]` | `clusterCelaFiles(java.io.File[] virtualCelFiles, int[][] pmCoordinates)`             Clusters cela files, extracts PM intensities, median normalizes. |
| `void` | `clusterNormalizedFloatArrayFiles()`             Clusters float[] files, does not median normalize |
| `java.io.File[]` | `clusterVirtualCelFiles(java.io.File[] virtualCelFiles, int[][] pmCoordinates)`             Performs actual clustering for CelFileQualityControl app. |
| `boolean` | `displayClusterPlot()` |
| `double[]` | `findBestPair()`             Takes an ArrayList of VCluster and finds the best pair based on a PearsonCorrelationCoefficient. |
| `java.util.ArrayList` | `getClusters()` |
| `Cluster` | `getMegaCluster()` |
| `double` | `getMinimalCorrelationCoefficient()` |
| `java.io.File` | `getPngResultFile()` |
| `java.lang.String` | `getTitle()` |
| `static void` | `main(java.lang.String[] args)` |
| `void` | `mergeClusters(double[] ccBestPair)`             Merges Clusters from best pair notes whether they have a bad cc. |
| `static void` | `printDocs()` |
| `void` | `processArgs(java.lang.String[] args)`             This method will process each argument and assign new varibles |
| `boolean` | `savePNG()` |
| `void` | `setDisplayClusterPlot(boolean displayClusterPlot)` |
| `void` | `setPngResultFile(java.io.File pngResultFile)` |
| `void` | `setSavePNG(boolean savePNG)` |
| `void` | `setTitle(java.lang.String panelTitle)` |

**Methods inherited from class java.lang.Object**

`equals, getClass, hashCode, notify, notifyAll, toString, wait, wait, wait`

**Constructor Detail**

### HierarchicalClustering

```
public HierarchicalClustering(java.lang.String[] args)
```

:   For stand alone use.

---


### HierarchicalClustering

```
public HierarchicalClustering(double minRValue,
                              int maxSizeToFlagCluster,
                              java.lang.StringBuffer results,
                              java.io.File pngResultFile,
                              boolean displayClusterPlot)
```

:   For integration with CelFileQualityControl App.

---


### HierarchicalClustering

```
public HierarchicalClustering(java.io.File[] floatArrayFiles)
```

:   For integration with Correlate App. Fires stand alone settings using the provided serialized float[] files.

---


### HierarchicalClustering

```
public HierarchicalClustering(java.io.File[] floatArrayFiles,
                              java.io.File pngResultFile)
```

:   For integration with CelProcessor.


**Method Detail**

### cluster

```
public void cluster()
```

---


### clusterVirtualCelFiles

```
public java.io.File[] clusterVirtualCelFiles(java.io.File[] virtualCelFiles,
                                             int[][] pmCoordinates)
```

:   Performs actual clustering for CelFileQualityControl app.

---


### clusterCelaFiles

```
public java.io.File[] clusterCelaFiles(java.io.File[] virtualCelFiles,
                                       int[][] pmCoordinates)
```

:   Clusters cela files, extracts PM intensities, median normalizes. Returns the names of virtual cel files with
    bad correlation coefficients.

---


### clusterNormalizedFloatArrayFiles

```
public void clusterNormalizedFloatArrayFiles()
```

:   Clusters float[] files, does not median normalize

---


### breakOutClusters

```
public void breakOutClusters()
```

---


### mergeClusters

```
public void mergeClusters(double[] ccBestPair)
```

:   Merges Clusters from best pair notes whether they have a bad cc.

---


### findBestPair

```
public double[] findBestPair()
```

:   Takes an ArrayList of VCluster and finds the best pair based on a
    PearsonCorrelationCoefficient. Returns a double[3]{cc, indexOne, indexTwo}.
    The HashMap contains calculated ccs. These will be used if the same comparison is made again.

---


### processArgs

```
public void processArgs(java.lang.String[] args)
```

:   This method will process each argument and assign new varibles

---


### printDocs

```
public static void printDocs()
```

---


### main

```
public static void main(java.lang.String[] args)
```

---


### getClusters

```
public java.util.ArrayList getClusters()
```

---


### getMegaCluster

```
public Cluster getMegaCluster()
```

---


### getMinimalCorrelationCoefficient

```
public double getMinimalCorrelationCoefficient()
```

---


### savePNG

```
public boolean savePNG()
```

---


### setSavePNG

```
public void setSavePNG(boolean savePNG)
```

---


### getPngResultFile

```
public java.io.File getPngResultFile()
```

---


### setPngResultFile

```
public void setPngResultFile(java.io.File pngResultFile)
```

---


### getTitle

```
public java.lang.String getTitle()
```

---


### setTitle

```
public void setTitle(java.lang.String panelTitle)
```

---


### displayClusterPlot

```
public boolean displayClusterPlot()
```

---


### setDisplayClusterPlot

```
public void setDisplayClusterPlot(boolean displayClusterPlot)
```


---


|  |  |  |
| --- | --- | --- |
| **Overview** \| **Package** \| **Class** \| **Use** \| **Tree** \| **Deprecated** \| **Index** \| **Help** | |  |
| **PREV CLASS**   NEXT CLASS | **FRAMES**    **NO FRAMES**     **All Classes** |  |
| SUMMARY: NESTED \| FIELD \| CONSTR \| METHOD | DETAIL: FIELD \| CONSTR \| METHOD |  |


---
